# Respiratory motion tracking of spine stereotactic radiotherapy in prone position

**DOI:** 10.1002/acm2.13910

**Published:** 2023-01-17

**Authors:** Kazufusa Mizonobe, Hiroaki Akasaka, Kazuyuki Uehara, Yuya Oki, Masao Nakayama, Shuhei Tamura, Yoshiki Munetomo, Katsumaro Kubo, Hiroki Kawaguchi, Aya Harada, Hiroshi Mayahara

**Affiliations:** ^1^ Division of Radiation Oncology Kobe Minimally Invasive Cancer Center Chuo‐ku, Kobe Hyogo Japan; ^2^ Department of Chemical Engineering The University of Melbourne, The University of Melbourne Grattan Street Parkville Victoria Australia; ^3^ Division of Radiation Oncology Kobe University Graduate School of Medicine Chuou‐ku, Kobe Hyogo Japan; ^4^ Division of Radiation Therapy Kita‐Harima Medical Center Ono Hyogo Japan; ^5^ Division of Radiological Technology Kobe Minimally Invasive Cancer Center Chuo‐ku, Kobe Hyogo Japan

**Keywords:** prone position, SBRT, spinal tumor, tumor‐tracking

## Abstract

**Purpose:**

The CyberKnife system is a specialized device for non‐coplanar irradiation; however, it possesses the geometric restriction that the beam cannot be irradiated from under the treatment couch. Prone positioning is expected to reduce the dose to normal lung tissue in spinal stereotactic body radiotherapy (SBRT) owing to the efficiency of beam arrangement; however, respiratory motion occurs. Therefore, the Xsight spine prone tracking (XSPT) system is used to reduce the effects of respiratory motion. The purpose of this study was to evaluate the motion‐tracking error of the spine in the prone position.

**Materials and Methods:**

Data from all 25 patients who underwent spinal SBRT at our institution between April 2020 and February 2022 using CyberKnife (VSI, version 11.1.0) with the XSPT tracking system were retrospectively analyzed using log files. The tumor motion, correlation, and prediction errors for each patient were examined. Furthermore, to assess the potential relationships between the parameters, the relationships between the tumor‐motion amplitudes and correlation or prediction errors were investigated using linear regression.

**Results:**

The tumor‐motion amplitudes in each direction were as follows: superior–inferior (SI), 0.51 ± 0.39 mm; left–right (LR), 0.37 ± 0.29 mm; and anterior–posterior (AP), 3.43 ± 1.63 mm. The overall mean correlation and prediction errors were 0.66 ± 0.48 mm and 0.06 ± 0.07 mm, respectively. The prediction errors were strongly correlated with the tumor‐motion amplitudes, whereas the correlation errors were not.

**Conclusions:**

This study demonstrated that the correlation error of spinal SBRT in the prone position is sufficiently small to be independent of the tumor‐motion amplitude. Furthermore, the prediction error is small, contributing only slightly to the tracking error. These findings will improve the understanding of how to compensate for respiratory‐motion uncertainty in the prone position.

## 1 INTRODUCTION

Fractionated three‐dimensional conformal radiotherapy (3DCRT) has been used as the standard palliative treatment for painful bone metastases.^1^ However, randomized trials have demonstrated that palliative stereotactic body radiotherapy (SBRT) produces faster pain control for spinal metastases than 3DCRT.^2^ Recently, SBRT has been utilized in various treatment devices for metastatic bone tumors and oligometastatic tumors.^3^, ^4^

When irradiating a spinal tumor using CyberKnife (Accuray Incorporated, Sunnyvale, CA), Xsight spine tracking (XST) is employed to track the tumor continuously using the spine as a landmark.^5^, ^6^ The CyberKnife system is a specialized device for non‐coplanar irradiation, which can irradiate beams from various directions.^6^, ^7^ However, the geometrical characteristics of the device do not allow the beam to be irradiated from under the couch. In the supine position, the dose to normal tissue may be high for posterior targets such as the spine. On the other hand, it is reportedly possible to avoid this situation by using the prone position.^8^, ^9^ However, it has been reported that the prone position induces larger respiratory motion than the supine position.^10^

In cases in which such motion occurs (e.g., in the lungs), the Synchrony respiratory tracking system has been used for tumor tracking,^11^, ^12^, ^13^, ^14^, ^15^, ^16^, ^17^ and the tracking data can be retrieved as log files after irradiation. Xsight spine prone tracking (XSPT) enables real‐time tracking by combining the Synchrony system with XST to compensate for respiratory motion in the prone position. The tracking error resulting from using the Synchrony system in the supine position during treatments of the lung, liver, and pancreas has been studied,^13^, ^14^, ^15^, ^16^, ^17^ whereas that in the prone position has not been investigated. In addition, Hoogeman et al. demonstrated that the prone position results in more systematic and accidental errors than the supine position.^18^ Therefore, it is necessary to examine the XSPT motion‐tracking error of the spine in the prone position in detail.

The objective of this study was to reveal whether a correlation model could be successfully created in the prone position using the Synchrony treatment‐log files of 25 patients who underwent XSPT at our institution. Furthermore, we investigated whether the tracking error depends on the amount of tumor motion. These data can be used to improve understanding of how to compensate for respiratory motion uncertainty.

## 2 MATERIALS AND METHODS

### 2.1 Patients

This study was approved by our institutional ethics committee. Data from 25 patients who underwent SBRT at our institution between April 2020 and February 2022 using CyberKnife (VSI, version 11.1.0) with the XSPT system were analyzed retrospectively. The patients were treated with prescription doses of 21–35 Gy in 2–5 fractions (a total of 69 fractions), depending on the patient attributes. The treatment plans were formulated using a Precision treatment‐planning system (version 2.0.1.1; Accuray Incorporated). The new VOLO optimizer, which is included in the Precision system, is intended to provide better plan quality with shorter treatment times.^19^ The planning treatment time (image time interval: 30 s; patient setup time: 10 min) in this study was 34 ± 3.6 min. All treatment plans were designed using 2 or 3 fixed‐size collimators with nonisocentric delivery.

### 2.2 Treatment using XSPT system

**FIGURE 1:**
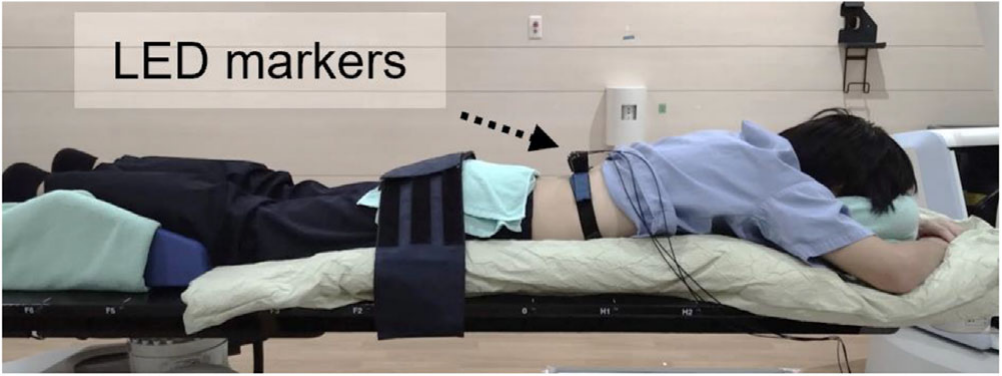
Prone‐setup example. Each patient was placed in the prone position. The pelvis was stabilized using a vacuum cushion, and the head was held fixed using a soft cushion. For treatment, a belt was used for additional stabilization. LED markers were placed on the back of the patient, where the waveform is prone to be as large as possible, and the respiratory waveforms were acquired

Each patient was placed in the prone position. The pelvis was stabilized using a vacuum cushion, and the head was held fixed using a soft cushion (Figure 1). We confirmed that the patients were comfortable and the position was painless. Three light‐emitting diode (LED) markers were placed on the back of the patient, where the waveform could be as large as possible, and respiratory waveforms were acquired. LED‐marker signals were obtained from a Synchrony camera mounted on the ceiling. Furthermore, the XSPT system compared orthogonal kilovolt live images of approximately three adjacent vertebrae with a pair of digitally reconstructed radiographs that were calculated from the planning computed‐tomography dataset to acquire the tumor position. The system built a correlation model, that is, it found a relationship between the position coordinates of the LED markers showing the respiratory motion and spine position. If a treatment fraction was interrupted because of unpredictable events such as excessive patient motion, a new correlation model was built by removing all existing data points, following the procedure of Hoogeman et al.^11^

### 2.3 Data collection

Five log files (Marker.log, Model.point.log, Modeler.log, Predictor.log, and ERsiData.log) were used.^13^ The CyberKnife system records data in two concentric coordinate systems (robot and patient). The data recorded by Modeler.log, Predictor.log, and ERsiData.log in robot coordinates were rotated by approximately 45° with respect to the horizontal plane and converted into patient coordinates.

### 2.4 Log file analysis

The correlation errors were analyzed using the ModelPoint.log file. The correlation error is defined as the difference between the tumor location found on radiographs and that calculated by utilizing the updated correlation model.^11^ The CyberKnife tracking system predicts the respiratory waveform after 115 ms and estimates the tumor location from the waveform position.^11^ The prediction error was calculated from the outputs of the predicted position (Predictor.log file) and the actual position (Modeler.log file) after 115 ms. In addition, to extract the prediction error during tracking, the ERsiData.log file, recording the offset value sent to the robot, was used.

The tumor positions were calculated from a correlation model and recorded in the Modeler.log file. Similarly, the positions of the LED markers were recorded in the Marker.log file, and the data for the axis perpendicular to the Synchrony camera were extracted. The motion amplitudes of the modeled tumor and LED markers were calculated per unit irradiation as the sum of the squares of the maximum distance moved in each direction while CyberKnife was tracking.

The mean, 95th percentile and 99th percentile values of the correlation and prediction errors were determined. The total error was calculated using the root‐square sum of the correlation and prediction errors in each direction following the procedure presented in a previous study.^14^

### 2.5 Statistical analysis

Statistical analyses were performed using EZR software (Saitama Medical Center, Saitama Medical University, Saitama, Japan; version 1.55).^20^ The data were calculated for each patient, and the overall mean ± SD for all patients was found. The Student's *t*‐test was used to assess the difference in the correlation error depending on the site (thoracic spine or lumbar spine). Linear‐regression analyses between the tumor‐motion amplitudes and the correlation or prediction errors were performed. Spearman's rank correlation coefficient *r* was used to assess the potential relationships between the parameters. The Bonferroni correction was applied to the critical *p*‐value to correct for multiple comparisons; a *p*‐value < 0.01 was considered statistically significant.

## 3 RESULTS

### 3.1 Tumor‐motion and LED‐marker‐motion amplitudes

In this work, 69 treatment fractions of 25 patients were studied. Table 1 summarizes the patient characteristics, including the tracking errors, number of fractions, and tumor‐motion and LED‐marker‐motion amplitudes. As shown in Table 2, the mean values of the tumor‐motion amplitudes in each direction were as follows: superior–inferior (SI), 0.51 ± 0.39 mm; left–right (LR), 0.37 ± 0.29 mm; anterior–posterior (AP), 3.43 ± 1.63 mm. The motion amplitude of the LED markers was 4.75 ± 1.97 mm.

**TABLE 1:** Summary of patient characteristics and tracking errors

Patient	Align center	M/F	fr	Error_Corr (mm)	SD	Error_Pred (mm)	SD	Tumor motion (mm)	LED motion (mm)
1	Th3	F	2	0.41	0.30	0.01	0.01	0.82	3.76
2	L3	F	2	0.32	0.20	0.06	0.06	4.15	3.39
3	Th9	F	3	0.51	0.27	0.04	0.04	2.67	3.40
4	Th11	F	2	0.53	0.24	0.05	0.05	2.55	2.72
5	Th10	F	2	0.44	0.33	0.05	0.06	2.59	3.14
6	Th7	M	3	0.83	0.60	0.06	0.08	3.09	4.31
7	Th11	M	2	0.70	0.31	0.10	0.12	4.88	6.32
8	Th8	F	2	0.79	0.66	0.02	0.02	1.34	4.28
9	L5	F	2	0.77	0.59	0.05	0.05	3.42	4.27
10	Th6	M	3	0.88	0.45	0.05	0.05	2.38	3.99
11	Th11	M	3	0.86	0.47	0.07	0.07	4.41	6.16
12	L4	M	3	0.47	0.25	0.06	0.07	3.41	5.25
13	Th10	M	5	0.93	0.62	0.10	0.11	6.83	4.99
14	Th4	F	3	0.71	0.50	0.03	0.03	2.05	6.56
15	Th7	F	3	0.73	0.41	0.05	0.05	4.07	9.41
16	Th12	M	3	0.61	0.34	0.06	0.06	2.52	2.95
17	L2	M	3	0.74	0.54	0.05	0.05	2.73	2.89
18	Th12	M	2	0.82	0.32	0.09	0.09	5.47	7.89
19	Th11	F	3	0.47	0.24	0.05	0.06	4.25	5.72
20	L1	F	3	0.49	0.22	0.06	0.07	4.68	5.30
21	L1	F	3	0.60	0.30	0.06	0.07	3.87	4.22
22	L3	F	3	0.34	0.20	0.06	0.07	3.59	4.24
23	Th9	F	3	0.73	0.41	0.03	0.03	2.01	4.17
24	L3	F	3	0.47	0.33	0.05	0.05	3.41	3.82
25	L3	M	3	0.27	0.17	0.08	0.10	3.86	4.21

Abbreviations: Error_Corr, absolute mean correlation error in the radial direction; Error_Pred, absolute mean prediction error in the radial direction; F, female; fr, number of fractions; L, lumbar spine; LED motion, mean LED‐marker‐motion amplitude; M, male; Th, thoracic spine; Tumor motion, mean tumor‐motion amplitude.

**TABLE 2:** Summary of tumor‐motion and LED‐marker‐motion amplitudes

	Tumor‐motion			
	SI	LR	AP	LED‐marker motion
Mean (mm)	0.51	0.37	3.43	4.75
SD	0.39	0.29	1.63	1.97

Abbreviations: AP, anterior–posterior; LR, left–right; SI, superior–inferior.

### 3.2 Correlation and prediction errors

**FIGURE 2:**
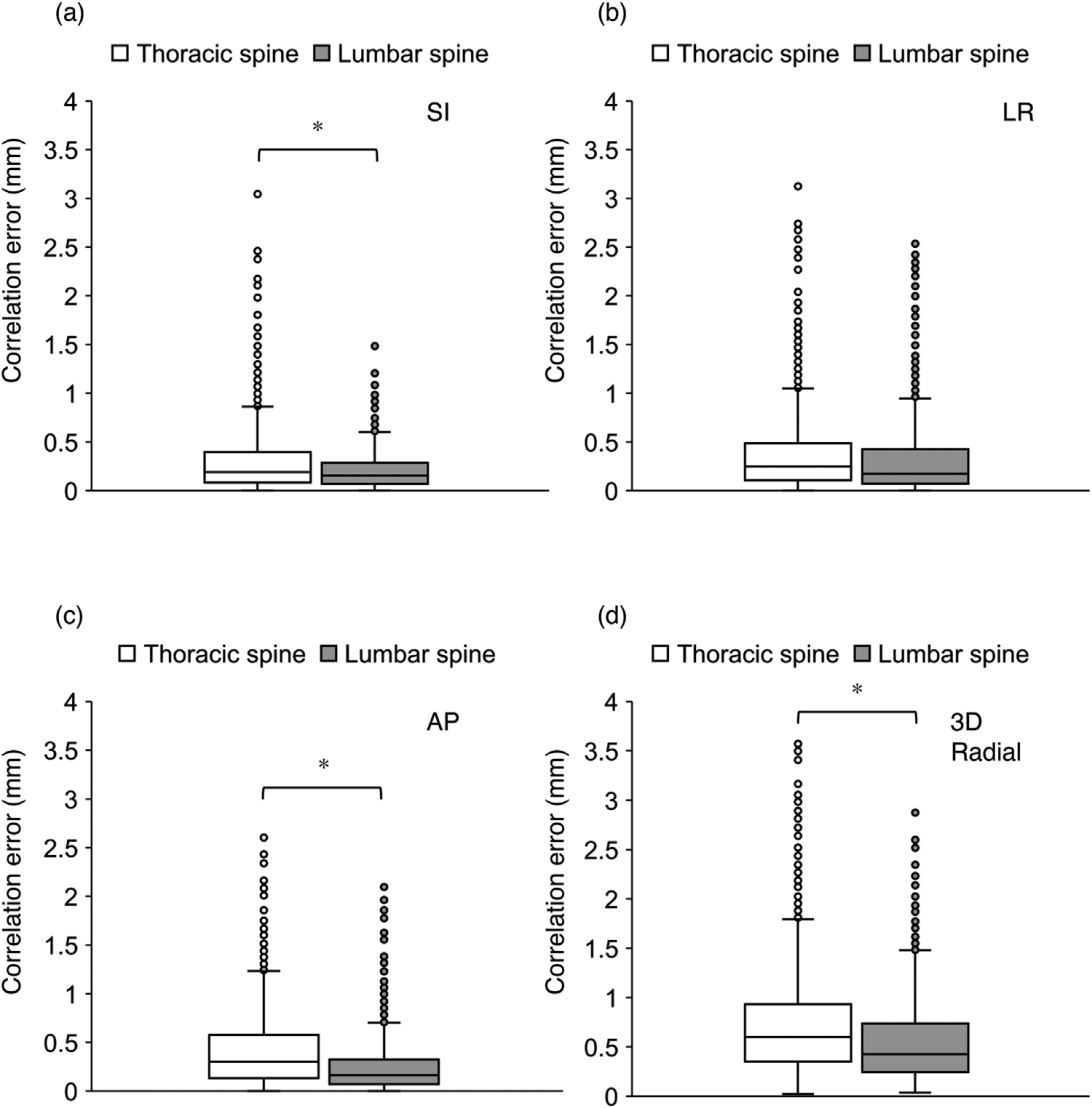
Boxplots of the correlation errors at the thoracic and lumbar spine in the (a) superior–inferior, (b) left–right, (c) anterior–posterior, and (d) three‐dimensional radial directions. AP, anterior–posterior; LR, left–right; SI, superior–inferior; 3D radial, three‐dimensional radial; *p < 0.01

The correlation errors of the thoracic and lumbar spine were evaluated, as shown in Figure 2. The correlation error in the latter was significantly smaller than that in the former in the AP, SI, and 3D radial directions (*p* < 0.01).

**FIGURE 3:**
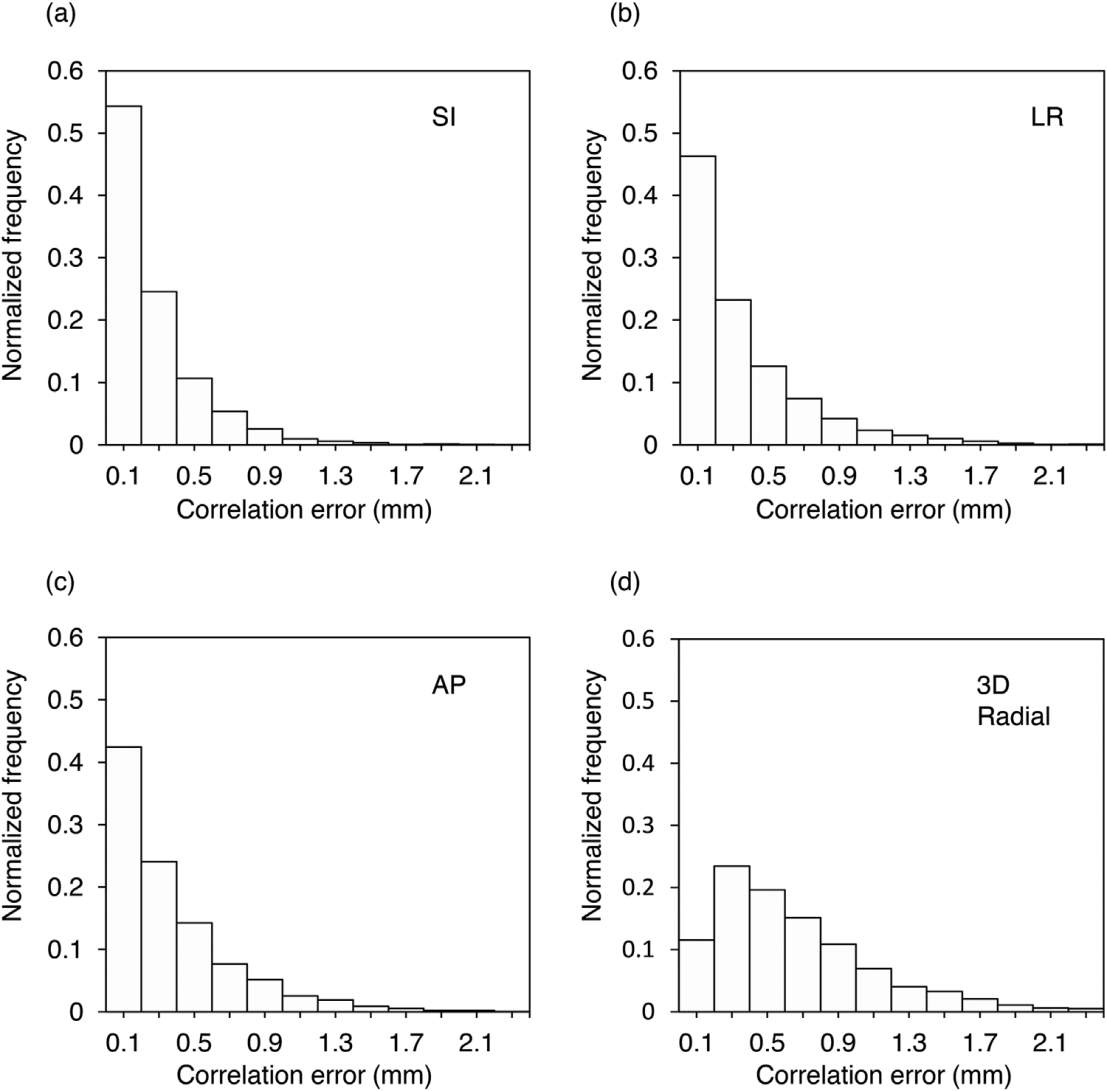
Histograms of the absolute translational correlation errors in the (a) SI, (b) LR, (c) AP, and (d) 3D radial directions for all patients and treatment fractions. The abbreviations are identical to those in Figure 2

Table 3 shows the absolute mean value, SD, 95th percentile value, and 99th percentile value of the correlation and prediction errors for all the patients. The overall mean value of the correlation error in the 3D radial direction was 0.66 ± 0.48 mm and the prediction error was 0.06 ± 0.07 mm. The prediction error had almost no effect on the total error. The histogram analysis of the correlation errors for all patients is displayed in Figure 3; the shape of the histogram was almost the same in each direction.

**TABLE 3:** Summary of the correlation and prediction errors for 25 patients

	Correlation error				Prediction error				Total error			
	SI	LR	AP	3D radial	SI	LR	AP	3D radial	SI	LR	AP	3D radial
Absolute mean (mm)	0.27	0.35	0.36	0.66	0.01	0.01	0.06	0.06	0.27	0.35	0.37	0.67
SD	0.28	0.37	0.36	0.48	0.01	0.01	0.07	0.07	0.28	0.37	0.36	0.49
95^th^ percentile (mm)	0.80	1.10	1.10	1.60	0.03	0.02	0.18	0.18	0.80	1.10	1.11	1.61
99^th^ percentile (mm)	1.31	1.67	1.60	2.27	0.06	0.05	0.34	0.34	1.31	1.67	1.64	2.30

Abbreviations: 3D, three‐dimensional; AP, anterior–posterior; LR, left–right; SI, superior–inferior.

### 3.3 Prediction error in case of the disturbed breathing

**FIGURE 4:**
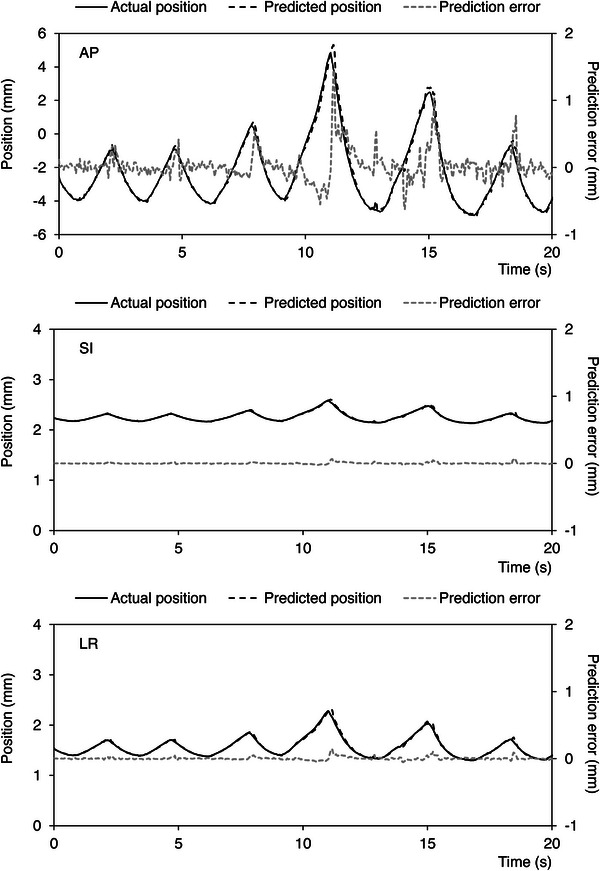
Results for Case 7. Actual position of the tumor (solid line), predicted position of the tumor (dashed line), and prediction error (dotted line) (secondary Y‐axis) in each direction. The predicted and actual positions are almost the same for most respiratory cycles. As soon as the amplitude changes, the prediction errors increase, particularly for the AP direction. The abbreviations are identical to those in Figure 2

Each patient in this study breathed freely during treatment, which introduced an unpredictable respiratory motion into the data. Figure 4 shows an example (Case 7) of the actual and predicted positions of the tumor and the prediction error (secondary Y‐axis). In the AP direction, a peak in the prediction error near 2 mm was observed, which corresponded to a notable breathing disturbance that prevented accurate prediction. However, the prediction error in each direction was almost negligible during stable breathing because the disturbed breathing was momentary.

### 3.4 Correlations between tumor‐motion amplitude and tracking error

**FIGURE 5:**
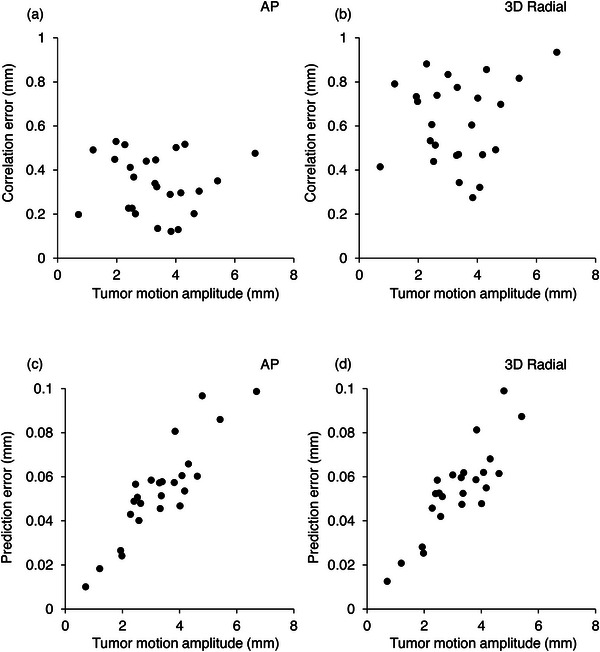
(a,b) Correlation errors as functions of tumor‐motion amplitude for the (a) AP and (b) 3D radial directions. (c,d) Prediction error as a function of tumor‐motion amplitude for the (c) AP and (d) 3D radial directions. The abbreviations are identical to those in Figure 2

The tumor‐motion amplitude was examined to determine its effects on the errors. Figure 5 shows the correlation and prediction errors as functions of the tumor‐motion amplitude in the AP and 3D radial directions, respectively. Table 4 lists the results of the regression analysis. No relation between the correlation errors in each direction and tumor‐motion amplitude was observed. In contrast, the prediction errors in each direction were strongly correlated with the tumor‐motion amplitude (SI, *r* = 0.84; LR, *r* = 0.85; AP, *r* = 0.85; 3D radial, *r* = 0.82; *p* < 0.01). However, as shown in Figure 5, the prediction error was less than 0.1 mm.

**TABLE 4:** Regression analysis results of the correlation and prediction errors

	Spearman *r* value	
Directions	Tumor amplitude vs. correlation error	Tumor amplitude vs. prediction error
SI	0.09	0.84*
LR	0.13	0.85*
AP	–0.16	0.85*
3D radial	0.05	0.82*

*Note*: The abbreviations are identical to those in Table 3.

* *p* < 0.01.

## 4 DISCUSSION

In this investigation, the log files of the XSPT system were analyzed to reveal whether a correlation model could be successfully created with the patient in the prone position. To our knowledge, no previous study has examined the motion‐tracking error of the spine in the prone position in detail. Therefore, our results provide important information with margins for designing spinal SBRT in the prone position. The tracking errors observed in this research are less than those in previous studies in which the supine position was used.^13^, ^14^, ^15^ For example, Winter et al. analyzed patients treated for liver cancer and reported mean correlation and prediction errors of 1.68 ± 1.13 mm and 0.26 ± 0.13 mm, respectively.^14^ These results may be attributed to the fact that the amplitude of tumor motion was greater than that observed in our study. The correlation errors are generally correlated with the tumor‐motion amplitude as reported by Nakayama et al.^15^ Liu et al. demonstrated that respiration‐induced motion in the prone position of lower thoracic spine is larger than those of other spine regions.^21^ The results shown in Table 1 (Cases 7, 11, 13, and 18) are consistent with those of Liu et al. The mean peak amplitude in the AP direction has been reported to be 1.27 ± 0.50 mm by Fürweger et al.,^22^ which is smaller than our result. This could be because the analysis was limited to the lower lumbar and sacral spine lesions in their study. Guy et al. reported that the maximum motion amplitude of Th12 was 4.5 mm in the prone position.^10^ Therefore, the respiratory‐induced motion should be addressed to reduce the tracking uncertainty. The amplitude of the LED markers in this study was 4.75 mm, which is smaller than those obtained in previous studies that used the supine position.^23^ It should be noted that it is possible that a smaller LED marker amplitude could increase the tracking error because the Synchrony system predicts the tumor location based on respiratory waveforms.^11^

The prone setup method has advantages as well as disadvantages. The prone position has the advantage that enables to reduce the entrance dose and the number of monitor units (MU) owing to the short path length of the photon beam.^8^, ^9^ The supine position tracking of the spine is known to achieve submillimeter precision, as reported by Fürweger et al.,^6^ which is higher than that of our results. A comparison of the planning study of spinal radiosurgery in supine and prone positions showed that when an additional target margin of 2 mm was added, the advantage of the prone position was reduced in most cases.^24^ However, their study did not consider the effect of gravity owing to the prone position because they rotated the images 180 degrees to create a prone plan. For example, the normal tissues‐to‐spinal cord separation in the prone position increases compared with the supine position.^25^ As a result, the dose of normal tissue near spinal cord could be efficiently reduced. In addition, Cox J. reported that there were no significant differences in comfort levels between treatment positions, prone or supine.^26^ On the other hand, Hideghéty K et al. reported that the supine position was considered more comfortable by the patients.^27^ For these reasons, the prone or supine position must be selected on a case‐by‐case basis. In this study, all patients tolerated prolonged treatments in prone position reasonably well; therefore, the tracking errors were sufficiently small, as shown in Table 3.

The next aspect to consider is body motion. Previous studies have reported that the intrafraction‐motion error was larger in the prone position than in the supine position.^8^, ^18^ This characteristic suggests that the outliers in Figure 2 were caused by excessive motion. Because the correlation error increases when patient motion occurs, Hoogeman et al.^11^ suggested that frequent checking and updating of the correlation model is necessary to reduce the effects of patient motion. Furthermore, according to previous studies, the cervical and upper thoracic spine experience more intrafraction motion than the lumbar spine because the cervical spine has greater mobility.^28^ Our data are consistent with these results, as the correlation error increased slightly in the thoracic spine.

The need for Synchrony tracking in the prone position is evident because the margins of spinal‐cord planning organ‐at‐risk volumes and metastatic‐bone‐tumor planning target volumes (PTVs) in spinal SBRT are generally extremely small, as reported in the literature.^3^, ^29^, ^30^ For example, Furuya et al. defined the PTV as the clinical target volume with a uniform expansion of 2 mm.^3^ As listed in Table 2, the average tumor‐motion amplitude in the AP direction was 3.43 mm, which was larger than the PTV margin. Thus, a concern exists that the tumor will not be irradiated correctly if Synchrony tracking is not applied. However, by using the XSPT system, the correlation error suggests that the model error was small regardless of the tumor‐motion amplitude, as shown in Figure 5b. In contrast, the prediction error was strongly correlated with the tumor‐motion amplitude, as reported following previous studies that gave results consistent with ours^14^, ^15^; however, the prediction error was very small, contributing only slightly to the tracking error. Therefore, the XSPT system is considered satisfactory for spinal SBRT in the prone position.

Our study possesses a few limitations. First, XSPT tracks only translational motion, not the rotational pitch, yaw, or roll motion for each respiratory phase; however, the operator can confirm in advance that the rotational motions are small (e.g., within 1°). Because the XST region of interest typically includes only three adjacent vertebral bodies,^31^ the effects of rotational errors on the tracking accuracy are considered small. Second, an end‐to‐end (E2E) test method for XSPT has not been established. An E2E test should be conducted for each track to evaluate the targeting error.^32^

## 5 CONCLUSIONS

In this study, we analyzed the correlation and prediction errors of 25 patients who underwent SBRT with the XSPT system. The correlation and prediction errors obtained in this investigation were significantly smaller than the tracking errors previously reported for other organs. Our results provide important information for assessing the uncertainty of the Synchrony respiratory‐tracking system in the prone position.

## AUTHOR CONTRIBUTIONS

Kazufusa Mizonobe, Hiroaki Akasaka, Kazuyuki Uehara, Yuya Oki, Masao Nakayama, Katsumaro Kubo, Hiroki Kawaguchi, Aya Harada, and Hiroshi Mayahara were involved in study design and data interpretation. Kazufusa Mizonobe, Shuhei Tamura, and Yoshiki Munetomo were involved in data acquisition and analysis. All authors critically revised the report, commented on drafts of the manuscript, and approved the final report.

## CONFLICT OF INTEREST

The authors declare that there are no conflicts of interest.

## Data Availability

Research data are stored in an institutional repository and will be shared upon request to the corresponding author.
